# Defining a diaphragm-focused and patient-acceptable inspiratory muscle training (IMT) load in difficult to wean, mechanically ventilated patients: a protocol for a prospective, mixed methods study

**DOI:** 10.1136/bmjresp-2026-004168

**Published:** 2026-06-24

**Authors:** Timothy O Jenkins, Mike I Polkey, David McWilliams, Cherry Kilbride, Vicky MacBean

**Affiliations:** 1 Rehabilitation and Therapies Department, Royal Brompton and Harefield Hospitals, Heart, Lung and Critical Care Group, Guy's and St Thomas’ NHS Foundation Trust, London, UK; 2 College of Health, Medicine and Life Sciences, Department of Health Sciences, Brunel University of London, London, UK; 3 Department of Respiratory Medicine, Royal Brompton and Harefield Hospitals, HLCC Group, Guy's and St Thomas’ Hospitals NHS Trust, London, UK; 4 Centre for Care Excellence, Coventry University, Coventry, UK; 5 Critical Care, University Hospitals Coventry and Warwickshire NHS Trust, Coventry, UK; 6 Therapy Services, Royal Free London NHS Foundation Trust, London, UK

**Keywords:** Exercise, Respiratory Muscles

## Abstract

**Introduction:**

Diaphragm dysfunction is associated with failure to wean from mechanical ventilation. Inspiratory muscle training (IMT) may help improve outcomes for difficult-to-wean patients, yet the optimal approach remains unknown. High load IMT causes a training effect on the extra-diaphragmatic inspiratory muscles, but does not improve weaning success, questioning the utility of frequently cited high intensity IMT loads. Additionally, patients may find IMT unpleasant and distressing due to dyspnoea experienced during IMT. The aim of this study is to define a diaphragm-focused IMT load which is acceptable to patients.

**Methods:**

This study is designed as a prospective, mixed methods study. Forty difficult to wean, tracheostomised patients on mechanical ventilation will perform IMT at 15, 30, 45 and 60% of their maximal inspiratory pressure (PImax) in a randomised order using a tapered flow resistive loading device (POWERBreathe KH2, POWERBreathe, UK). Electrical activity of the diaphragm will be measured using an oesophageal multipair catheter (NAVA, Getinge, Sweden); extra-diaphragmatic (parasternal, scalene and sternocleidomastoid) muscle activity will be measured using surface electromyography. Patient experience of each load will be explored by measuring perceived breathing difficulty and unpleasantness using 0–10 numeric rating scales, verbal or written communication techniques in line with the participants' communication ability, and/or bespoke communication boards co-designed with the study patient and public involvement (PPI) group. Quantitative and qualitative data will be integrated to inform a diaphragm-focused, patient-acceptable IMT load.

**Ethics and dissemination:**

Ethical approval was obtained from Wales Research Ethics Committee 4 (Reference 25/WA/0242) on 30 October 2025. Results from this study will be presented at scientific meetings and published in peer-reviewed journals.

**Trial registration number:**

NCT07256821.

**WHAT IS ALREADY KNOWN ON THIS TOPIC**

- Previous work evaluating inspiratory muscle training (IMT) in mechanically ventilated patients has shown variable effects on strength and clinical outcomes, with training regimes often lacking a clear physiological basis and studies disregarding the patient experience.

**WHAT THIS STUDY ADDS**

- This prospective, mixed methods study will examine the electrical activity of the diaphragm and selected extra-diaphragmatic muscles during four IMT intensities. Patient experience of IMT will be explored contemporaneously using a bespoke range of communication approaches.

**HOW THIS STUDY MIGHT AFFECT RESEARCH, PRACTICE OR POLICY**

- Quantitative and qualitative data will be integrated to determine an IMT load that focuses on diaphragm activation and is psychologically acceptable to patients.
- The results of this study will be used to inform the design of a feasibility randomised controlled trial.

## Introduction

 Critically ill patients undergoing mechanical ventilation (MV) frequently develop diaphragm dysfunction. Diaphragm dysfunction is present in 63–80% of patients mechanically ventilated for over 48 hours^1 2^ and is associated with prolonged ventilation, increased mortality and readmission to intensive care (ICU).^13^,^7^ Patients who require prolonged ventilation (‘difficult to wean’ patients) have increased long-term cognitive and functional impairments, poorer post-discharge survival,^5 8^ occupy a disproportionate number of ICU bed days and have greater healthcare costs.^9 10^

Inspiratory muscle training (IMT) has been proposed as a treatment strategy to improve outcomes in difficult to wean patients by improving the strength and endurance of the diaphragm through the application of resistance during inspiration.^11^ IMT has been shown to improve overall volitional inspiratory muscle strength (PImax) in many studies; however, three systematic reviews have concluded there is insufficient evidence to confirm whether it has a positive effect on clinical outcomes such as weaning duration and ICU and hospital length of stay.^12^,^14^ Furthermore, a large randomised controlled trial (RCT) recently conducted by Van Hollebeke *et al* found no significant differences in PImax or weaning success following sham versus high load IMT in difficult to wean patients.^15^

One reason why studies may fail to consistently find an effect with IMT is due to the heterogeneity of training approaches employed^16 17^; training regimes vary widely across the literature employing endurance or strength-based training at loads set anywhere between 20 and 50% of the patient’s PImax, often increased to the maximum tolerable by the patient.^1518^,^23^ Most studies lack a clear physiological basis for the training regime chosen. Importantly, it is specific diaphragm weakness that is associated with weaning failure in mechanically ventilated patients, not overall inspiratory strength. IMT will not only target the diaphragm but also the extra diaphragmatic inspiratory muscles, to a greater extent as the load increases.^24^ High load IMT is frequently used in difficult to wean patients,^15 19 21 22^ causing a training effect on the scalene and sternocleidomastoid muscles,^25^ but does not improve weaning success,^15^ perhaps unsurprising as extra-diaphragmatic muscles are not useful for sustained ventilatory effort in mechanically ventilated patients.^16 26 27^ As IMT load increases, so does the metabolic demand on the patient, for possibly no physiological benefit.^28^

It has been proposed that a high load, interval approach to IMT is ideal for mechanically ventilated patients to briefly load the inspiratory muscles.^29^ However, high load IMT (breathing forcefully) may cause patients to experience dyspnoea, a sensation consistently associated with the fear of dying in mechanically ventilated patients.^30 31^ Patient and public involvement (PPI) in the planning of this project identified that high load IMT can be unpleasant and distressing. These factors may explain the variable completion rates of planned high load IMT sessions reported in the literature (between 17 and 88%), with patient refusal or fatigue commonly cited as the reasons for non-adherence.^15 19 22^ An IMT load that patients find acceptable may help reduce the negative short and long-term effects of dyspnoea, which could be caused by IMT, such as immediate distress^31 32^ or longer term post-traumatic stress disorder.^33^ In turn, an acceptable load may reduce refusal rates, enhancing the efficacy of the intervention.

There are calls for the optimisation of the IMT stimulus in difficult to wean patients using the principles of muscle physiology and exercise prescription^16 17 25^; hence, this study aims to define not only a physiologically informed IMT load which focuses on training the diaphragm but also, importantly, a load which is acceptable to patients.

The objectives of this study are as follows: (1) Measure diaphragm and extra diaphragmatic (parasternal intercostal, scalene and sternocleidomastoid) inspiratory muscle activity using electromyography (EMG) during four different IMT loads in tracheostomised, difficult to wean, patients. (2) Explore the experience of these varied IMT loads using difficulty and unpleasantness numeric rating scales (NRS) and qualitative methods in line with the participants’ communication ability. (3) Integrate quantitative and qualitative data to determine a diaphragm-focused and patient-acceptable IMT load.

## Methods and analysis

### Patient and public involvement (PPI)

A PPI group was formed in the planning stages of this study, consisting of one relative of an ICU survivor and six ICU survivors, all of whom experienced prolonged mechanical ventilation in ICU. Five members had performed IMT when mechanically ventilated. The PPI group met with the research team on 12, 1-hour long occasions; their participation was fully remunerated in line with the NIHR payment guidance for public contributors.^34^

PPI was integral to the design of the study. Group members offered their lived experience of IMT when mechanically ventilated, including the following recollections: ‘mild training almost pleasant, extreme training like dying’, ‘they (IMT) were hard tasks, my chest felt tight and I felt very tired’, ‘not understanding what and why’, ‘recollections of breathing training formed hallucinations, dreams and nightmares’. Following this feedback, the study was changed from a physiological study measuring muscle activity only into a mixed methods study, reflecting the importance of patient experience when seeking the optimal IMT load.

The group understood the value of the research and felt they or their next of kin would consent to the study. The group advised for a longer period for qualitative data collection as they felt participants would be fatigued and breathless after IMT. They offered first-hand accounts of their changing cognition in ICU and suggested one to three research occasions per participant to improve the quality of the qualitative data and account for variable engagement. The group gave key words describing their experience of IMT or ventilator weaning (if they had not performed IMT). Over a period of six, hour-long PPI meetings, they co-designed the picture and word boards and numerical rating scale (NRS) anchors (online supplemental figures 1–6) that will be used to help participants describe how they experienced each IMT load. The group will help co-produce dissemination material and provide guidance on dissemination methods to reach wider patient communities.

### Trial design and setting

Prospective, mixed methods study that will be conducted on five medical, surgical and transplant ICUs within Guy’s and St Thomas’ NHS Foundation Trust, UK.

### Eligibility criteria

Patients will be eligible if they are mechanically ventilated via a tracheostomy; aged ≥18 years; co-operative and able to participate in IMT. Participants must be able to give informed consent or, in cases where the patient lacks capacity, surrogate approval will need to be granted by a personal consultee (a relative or friend). Exclusion criteria are as follows: facial or skull fracture, or a bleeding disorder, which would prohibit oesophageal catheter placement; undrained pneumothorax/pneumomediastinum; delirium or deep sedation that impedes the patient’s ability to participate in study procedures; pregnancy; unlikely to survive or attending consultant assessment that the patient is not appropriate.

### Recruitment

An electronic patient record (Epic Systems Corporation, Wisconsin, USA) will be used to screen for patients eligible for the study. Researchers will access this record daily to ensure eligible patients can be identified and approached about the possibility of participating in the study.

### Study procedures

#### Measurement of inspiratory muscle activity

Electrical activity of the diaphragm (EMGdia) will be measured using an oesophageal multipair catheter (NAVA, Getinge, Sweden), inserted according to manufacturer guidelines. The catheter will be inserted into the participant’s nose and passed into the stomach using the distance from the nasal bridge to the earlobe to xiphoid process measurement. The catheter will be connected to a ventilator (Servo-I, Getinge, Sweden), loaded with software (Servotracker, Getinge, Sweden), to verify catheter placement and to perform amplification and digital processing of EMGdia signals using the system’s proprietary algorithms. The Servo-I will only be used for acquisition of EMGdia data, not for mechanical ventilation.

Parasternal intercostal, scalene and sternocleidomastoid muscle activity will be measured using surface electromyography (sEMG). Skin will be prepared by shaving the area (if necessary) and lightly abrading the skin with Nuprep skin preparation gel (Weaver and Company, Aurora, USA) before wiping with an alcohol-impregnated wipe to remove sebum and dead skin cells before application of silver-silver-chloride electrodes. Parasternal intercostal EMG will be obtained with electrodes positioned over the second intercostal space bilaterally.^35^ Scalene and sternocleidomastoid electrodes will be positioned over the inferior portion of each muscle as previously described.^36^ EMG signals will be amplified (gain 1000) and band-pass filtered (20–1000 Hz with additional 50 Hz adaptive mains filter, (BioAmp amplifier, ADInstruments, Sydney, Australia)) and digitised (sampling frequency 2 kHz, PowerLab 16/35, ADInstruments, Sydney, Australia) before being displayed on a laptop computer for analysis.

Root-mean-squared values of EMG signals will be calculated in LabChart 7 (ADInstruments, Sydney, Australia) and Servotracker (Getinge, Sweden) and mean peak RMS EMG value per breath at each IMT load will be expressed as a ratio to that obtained while breathing during unsupported spontaneous breathing.

#### Airway pressure and flow

Respiratory flow (with derived volumes) will be measured continuously throughout the study protocol using a standard pneumotachograph (4700 series, Hans Rudolph Inc, Shawnee, USA) attached to a pressure transducer (Spirometer, ADInstruments, Sydney, Australia). A side port will measure pressure at the airway opening (MK1S, GM Instruments, Irvine, UK). Both signals will be digitised (sampling frequency 100 Hz, PowerLab 16/35, ADInstruments, Sydney, Australia) and displayed on LabChart7 software (ADInstruments, Sydney, Australia).

#### Maximal inspiratory pressure

PImax will be measured on each study occasion with the participant positioned in a semi-recumbent position in bed using a unidirectional valve (Hans Rudolph Inc, Shawnee, US) attached to the participant’s tracheostomy tube. The valve allows exhalation but occludes inspiration. In a method described by Marini and colleagues,^37^ participants will be encouraged to inhale repeatedly as forcefully as possible for 20 s. The valve will be connected to a pressure transducer (MK1S, GM Instruments, Irvine, UK), and data displayed on LabChart7 software. Measurement will be repeated at least three times, until three measures within 10% of each other are obtained,^38^ with a 2-min rest in between manoeuvres. The greatest peak pressure will be used for analysis.

#### Other assessments

Date of ICU admission, date of onset of mechanical ventilation, date of tracheostomy insertion, admission diagnosis, past medical history, Acute Physiology and Chronic Health Evaluation score (APACHE II) for the first 24 hours of admission, and Sequential Organ Failure Assessment (SOFA) score on each study occasion will be obtained from the patient’s electronic health record. Ventilation parameters (mode, Positive End Expiratory Pressure (PEEP); pressure support; peak inspiratory pressure; respiratory rate (set and spontaneous); tidal volume; minute volume; FiO_2_) and cardiovascular parameters (blood pressure, heart rate, vasopressor use) will be obtained on each study occasion. Airway occlusion pressure at 100 milliseconds (P0.1), and expiratory occlusion pressure (Pocc) will be measured on each study occasion using built-in functions on the participant’s ventilator.

#### Breathing conditions measured

Respiratory muscle activity, airway pressure and airway flow will be measured during the following breathing conditions: (1) spontaneous breathing supported by mechanical ventilation with pressure support, with settings maintained unchanged from those determined by the attending intensivist; (2) unsupported spontaneous breathing through tracheostomy after disconnection from mechanical ventilation; (3) during four IMT loads.

During spontaneous breathing supported by mechanical ventilation, the participant will receive no additional instructions from the researcher; measurements will be taken for 5 min. During unsupported spontaneous breathing and IMT, the participant will be informed that their breathing will feel harder. The researcher will count each of the ten breaths at each IMT load to guide the participant. Participants will be coached to exhale fully prior to each IMT breath and be given strong verbal encouragement to inhale forcefully against the applied resistance to generate tidal volume up to a comfortable deep inspiration at their own pace; no set breathing frequency will be imposed.

IMT will be performed using a tapered-flow resistive loading device (POWERbreathe KH2, POWERbreathe, Southam, UK). Participants will perform IMT at 15, 30, 45 and 60% of their maximal inspiratory pressure (PImax) in a randomised order (Sealed Envelope, Oxford, UK). The IMT loads chosen are based on preliminary data from healthy participants (study approved by Brunel University of London: reference 45378-MHR-Jan/2024-49666-3; manuscript in preparation)**,** where we found an increase in diaphragm activity with increasing IMT load, which, from 30–40% of PImax was accompanied by a proportionally greater increase in extra-diaphragmatic muscle activity. We have therefore chosen loads spanning the inflection point seen in healthy participants, with the aim of identifying a load which maximises diaphragm activity but which minimises accessory muscle activity.

#### Exploring the patient experiences and perspectives of IMT load

Participant experience of IMT will be explored after each load. As participants will have a range of communication abilities, the researchers will work with speech and language therapists and staff in the ICU to optimise participants’ ability to communicate and express their experience of IMT. Communication methods will include the following: inline speaking valve, above cuff vocalisation, electrolarynx, bespoke communication boards, facial expressions, written communication and mouthing.^39^,^41^ An indicative topic guide to guide enquiry was co-developed and tested with the project’s PPI group.

Perceived breathing difficulty and unpleasantness will be measured using 0–10 numeric rating scales, as previously described^31^ with anchor points informed by the study PPI group: “0=none”, “10=extreme” for breathing unpleasantness (online supplemental file 1), “0=easy”, “10=extremely difficult” for breathing difficulty (online supplemental figure 2).

In participants who can phonate or respond using mouthing or written communication, following completion of the difficulty numeric rating scale (online supplemental figure 2), the participant will be asked “can you explain in words what that number felt like?” Due to the complexity of needs in this population, the researcher will use a range of strategies to enhance the depth of enquiry, such as confirmatory paraphrasing and summarising back to participants to check understanding of their response.

In participants unable to engage in independent expression of feelings as detailed above and to supplement information gained by verbal, written or lip reading, bespoke communication boards will be used (online supplemental figures 3–6). Within this complex population, when factors such as difficulty in phonating, breathlessness, weakness or fatigue with written communication limit answers, the bespoke communication boards will help improve the depth of information obtained. Contextual information (eg, participant gestures and the environment) will be captured using field notes, audio and/or video recording (subject to optional participant consent for video).

Participants will perform all study procedures on up to three occasions over a 7-day period. Reasons for earlier withdrawal would include decannulation from tracheostomy, weaning from mechanical ventilation, withdrawal of consent, or death. Table 1 provides an overview of study interventions and assessments performed during the study period.

**Table 1:** Overview of study procedures

Study procedures	Study period	
	Day 0 (enrolment)	Each study occasion
Baseline characteristics	●	
SOFA score		●
Ventilation and cardiovascular parameters		●
Maximal inspiratory pressure (PImax)		●
Inspiratory muscle activity		●
Airway pressure and flow		●
Inspiratory muscle training		●
Perceived difficulty and unpleasantness (NRS)		●
Qualitative data enquiry		●

NRS, numerical rating scale; SOFA, sequential organ failure assessment.

### Data analysis plan

#### Quantitative

Data will be assessed for normality using histograms and the Shapiro-Wilk test. Baseline characteristics will be presented as mean (SD) or median (IQR). Mean peak root-mean-squared values of EMGdia, parasternal intercostal, scalene and sternocleidomastoid EMG for each load will be normalised as a ratio to that obtained during unsupported spontaneous breathing. For each individual muscle, the EMG activity between different IMT loads will be compared using a one-way ANOVA or Kruskal-Wallis test. To evaluate patterns of muscle activity between sEMG of the parasternal intercostal, scalene and sternocleidomastoid muscles and EMGdia, plots will be visually evaluated on Prism (GraphPad Software Inc, California, US) to identify the IMT load inducing maximum diaphragmatic contribution before the upward inflection point of accessory muscle activity.

#### Qualitative

Audio-recordings of qualitative data will be transcribed verbatim by a professional medical transcription company and managed through NVivo software (Lumivero, Denver, US). Qualitative data will be analysed using reflexive thematic analysis^42^; the depth and confidence of themes may be limited dependent on the data generated. Qualitative data will be contextualised by the inclusion of the picture and word boards and audio/video recordings.

#### Integration of quantitative and qualitative data

Following the separate analysis of the quantitative and qualitative data sets, the data will be integrated and presented in side-by-side joint displays.^43 44^ Side-by-side displays place quantitative and qualitative information alongside each other in parallel columns so that they can be directly compared and integrated for interpretations to be made. The display will highlight areas of agreement (convergence), partial agreement (complementary / overlap) or silence (non-overlap) to ascertain new insights beyond the information obtained from the separate quantitative and qualitative results.

### Sample size calculation

This is the first study of its kind to measure inspiratory muscle activity and explore patient experience during different IMT loads in mechanically ventilated patients. No formal sample size calculation has been performed; however, based on data from the healthy study data conducted by our group, and accounting for the anticipated heterogeneity in the ICU population, we hypothesise that studying 40 participants will allow adequate exploration across one to three study occasions.

### Data management

On all study documents, other than the signed consent form and/or personal consultee forms (which will be stored separate from study data in a locked filing cabinet in a secure office), the participant will be referred to by a unique study code. The document that links participant codes to personal details will be kept on a password protected file, accessible by the immediate research team only, separate from study data. The following data will be uploaded after each session to a password-protected folder on a secure NHS network drive which is backed up every 24 hours: pseudonymised quantitative data (held on LabChart or password protected files), field notes and written communication from qualitative data enquiry (scanned and uploaded); audio and video files of qualitative data enquiry.

### Adverse events and withdrawal

No serious adverse events have been reported with IMT in mechanically ventilated patients.^13 15^ Risks of oesophageal multipair catheter insertion are minimised by the exclusion criteria for the study. Other study measurements are low risk in nature. Any adverse events occurring during the study will be reported to the chief investigator and the attending clinician in ICU. Participants (or personal consultees on the patient’s behalf) can withdraw from the study at any time.

### Ethics and dissemination

Ethical approval was obtained from Wales Research Ethics Committee 4 (Reference 25/WA/0242) on 30 October 2025. Results from this study will be presented at scientific meetings and published in peer reviewed journals. One member of the PPI group will be invited to a national critical care conference to co-present the findings.

## Supplementary material

10.1136/bmjresp-2026-004168 online supplemental file 1

## Data Availability

Data sharing not applicable as no datasets generated and/or analysed for this study.

## References

[1] Dres M, Dubé B-P, Mayaux J (2017). Coexistence and Impact of Limb Muscle and Diaphragm Weakness at Time of Liberation from Mechanical Ventilation in Medical Intensive Care Unit Patients. Am J Respir Crit Care Med.

[2] Jung B, Moury PH, Mahul M (2016). Diaphragmatic dysfunction in patients with ICU-acquired weakness and its impact on extubation failure. Intensive Care Med.

[3] Goligher EC, Dres M, Fan E (2018). Mechanical Ventilation-induced Diaphragm Atrophy Strongly Impacts Clinical Outcomes. Am J Respir Crit Care Med.

[4] De Jonghe B, Bastuji-Garin S, Durand M-C (2007). Respiratory weakness is associated with limb weakness and delayed weaning in critical illness. Crit Care Med.

[5] Béduneau G, Pham T, Schortgen F (2017). Epidemiology of Weaning Outcome according to a New Definition. The WIND Study. Am J Respir Crit Care Med.

[6] Supinski GS, Callahan LA (2013). Diaphragm weakness in mechanically ventilated critically ill patients. Crit Care.

[7] Damuth E, Mitchell JA, Bartock JL (2015). Long-term survival of critically ill patients treated with prolonged mechanical ventilation: a systematic review and meta-analysis. Lancet Respir Med.

[8] Wilson ME, Barwise A, Heise KJ (2018). Long-Term Return to Functional Baseline After Mechanical Ventilation in the ICU. Crit Care Med.

[9] Zilberberg MD, Nathanson BH, Ways J (2020). Characteristics, Hospital Course, and Outcomes of Patients Requiring Prolonged Acute Versus Short-Term Mechanical Ventilation in the United States, 2014-2018. Crit Care Med.

[10] Hill AD, Fowler RA, Burns KEA (2017). Long-Term Outcomes and Health Care Utilization after Prolonged Mechanical Ventilation. Ann Am Thorac Soc.

[11] Bureau C, Van Hollebeke M, Dres M (2023). Managing respiratory muscle weakness during weaning from invasive ventilation. Eur Respir Rev.

[12] Vorona S, Sabatini U, Al-Maqbali S (2018). Inspiratory Muscle Rehabilitation in Critically Ill Adults. A Systematic Review and Meta-Analysis. Ann Am Thorac Soc.

[13] Elkins M, Dentice R (2015). Inspiratory muscle training facilitates weaning from mechanical ventilation among patients in the intensive care unit: a systematic review. J Physiother.

[14] McCormack E, McDonough S, Kelly YP (2025). What is the effect of measurable respiratory muscle training on respiratory muscle strength in mechanically ventilated adults in intensive care units? A systematic review and meta-analysis. Aust Crit Care.

[15] Van Hollebeke M, Poddighe D, Hoffman M (2025). Similar Weaning Success Rate with High-Intensity and Sham Inspiratory Muscle Training: A Randomized Controlled Trial (IMweanT). Am J Respir Crit Care Med.

[16] Ballesteros-Reviriego G, Arbillaga-Etxarri A, Martí JD (2024). Inspiratory Muscle Training: Back to Basics Must be the First Step?. *Archivos de Bronconeumología*.

[17] Shei R-J, Paris HL, Sogard AS (2021). Time to Move Beyond a “One-Size Fits All” Approach to Inspiratory Muscle Training. Front Physiol.

[18] Cader SA, Vale RG de S, Castro JC (2010). Inspiratory muscle training improves maximal inspiratory pressure and may assist weaning in older intubated patients: a randomised trial. J Physiother.

[19] Martin AD, Smith BK, Davenport PD (2011). Inspiratory muscle strength training improves weaning outcome in failure to wean patients: a randomized trial. Crit Care.

[20] Condessa RL, Brauner JS, Saul AL (2013). Inspiratory muscle training did not accelerate weaning from mechanical ventilation but did improve tidal volume and maximal respiratory pressures: a randomised trial. J Physiother.

[21] Sandoval Moreno LM, Casas Quiroga IC, Wilches Luna EC (2019). Efficacy of respiratory muscle training in weaning of mechanical ventilation in patients with mechanical ventilation for 48hours or more: A Randomized Controlled Clinical Trial. Med Intensiva (Engl Ed).

[22] Bissett BM, Leditschke IA, Neeman T (2023). Does mechanical threshold inspiratory muscle training promote recovery and improve outcomes in patients who are ventilator-dependent in the intensive care unit? The IMPROVE randomised trial. Aust Crit Care.

[23] Roceto Ratti L dos S, Marques Tonella R, Castilho de Figueir≖do L (2022). Inspiratory Muscle Training Strategies in Tracheostomized Critically Ill Individuals. Respir Care.

[24] Ward ME, Eidelman D, Stubbing DG (1988). Respiratory sensation and pattern of respiratory muscle activation during diaphragm fatigue. J Appl Physiol (1985).

[25] Van Hollebeke M, Poddighe D, Clerckx B (2022). High-Intensity Inspiratory Muscle Training Improves Scalene and Sternocleidomastoid Muscle Oxygenation Parameters in Patients With Weaning Difficulties: A Randomized Controlled Trial. Front Physiol.

[26] Dres M, Dubé B-P, Goligher E (2020). Usefulness of Parasternal Intercostal Muscle Ultrasound during Weaning from Mechanical Ventilation. Anesthesiology.

[27] Parthasarathy S, Jubran A, Laghi F (2007). Sternomastoid, rib cage, and expiratory muscle activity during weaning failure. J Appl Physiol (1985).

[28] Jenkins TO, MacBean V, Poulsen MK (2023). The metabolic cost of inspiratory muscle training in mechanically ventilated patients in critical care. Intensive Care Med Exp.

[29] Bissett B, Leditschke IA, Green M (2019). Inspiratory muscle training for intensive care patients: A multidisciplinary practical guide for clinicians. Aust Crit Care.

[30] Haugdahl HS, Storli SL, Meland B (2015). Underestimation of Patient Breathlessness by Nurses and Physicians during a Spontaneous Breathing Trial. Am J Respir Crit Care Med.

[31] Demoule A, Decavele M, Antonelli M (2024). Dyspnoea in acutely ill mechanically ventilated adult patients: an ERS/ESICM statement. Intensive Care Med.

[32] Karlsson V, Lindahl B, Bergbom I (2012). Patients’ statements and experiences concerning receiving mechanical ventilation: a prospective video-recorded study. Nurs Inq.

[33] Başoğlu M (2017). Effective management of breathlessness: a review of potential human rights issues. Eur Respir J.

[34] National Institute for Health and Care Research Payment guidance for researchers and professionals involving people in research. https://www.nihr.ac.uk/payment-guidance-researchers-and-professionals.

[35] MacBean V, Wheatley L, Lunt AC (2017). Respiratory load perception in overweight and asthmatic children. Respir Physiol Neurobiol.

[36] Jonkman AH, Warnaar RSP, Baccinelli W (2024). Analysis and applications of respiratory surface EMG: report of a round table meeting. Crit Care.

[37] Marini JJ, Smith TC, Lamb V (1986). Estimation of inspiratory muscle strength in mechanically ventilated patients: The measurement of maximal inspiratory pressure. J Crit Care.

[38] Laveneziana P, Albuquerque A, Aliverti A (2019). ERS statement on respiratory muscle testing at rest and during exercise. Eur Respir J.

[39] Bjerregaard Alrø A, Svenningsen H, Korvenius Nedergaard H (2024). Cognitive impairment in intensive care unit patients: A qualitative exploration through observations and interviews. Intensive Crit Care Nurs.

[40] Zaga CJ, Freeman-Sanderson A, Happ MB (2023). Defining effective communication for critically ill patients with an artificial airway: An international multi-professional consensus. Intensive Crit Care Nurs.

[41] Ten Hoorn S, Elbers PW, Girbes AR (2016). Communicating with conscious and mechanically ventilated critically ill patients: a systematic review. Crit Care.

[42] Braun V, Clarke V (2022). Thematic Analysis: A Practical Guide. Bpsptr.

[43] Creswell JW (2022). A concise introduction to mixed methods research.

[44] Fetters MD, Curry LA, Creswell JW (2013). Achieving integration in mixed methods designs-principles and practices. Health Serv Res.

