# Supplementary material for: Defining a diaphragm-focused and patient-acceptable inspiratory muscle training (IMT) load in difficult to wean, mechanically ventilated patients: a protocol for a prospective, mixed methods study
Source: BMJ Open Respir Res. 2026 Jun 24;13(1):e004168. doi: 10.1136/bmjresp-2026-004168 (PMC13295908; doi:10.1136/bmjresp-2026-004168)

Supplementary material

Supplementary figure 1: Unpleasantness numerical rating scale board.


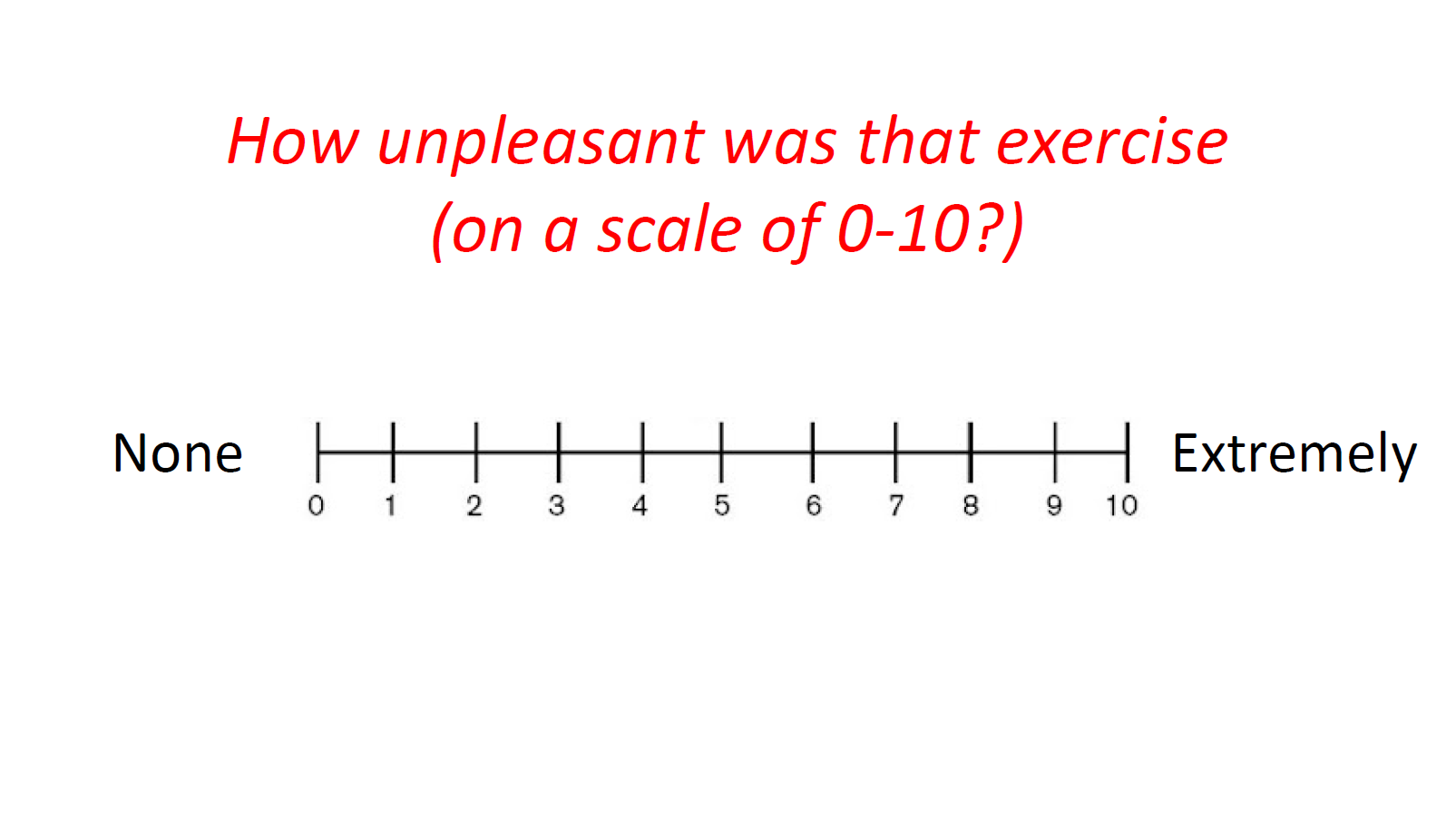


Supplementary figure 2: Difficulty numerical rating scale board.


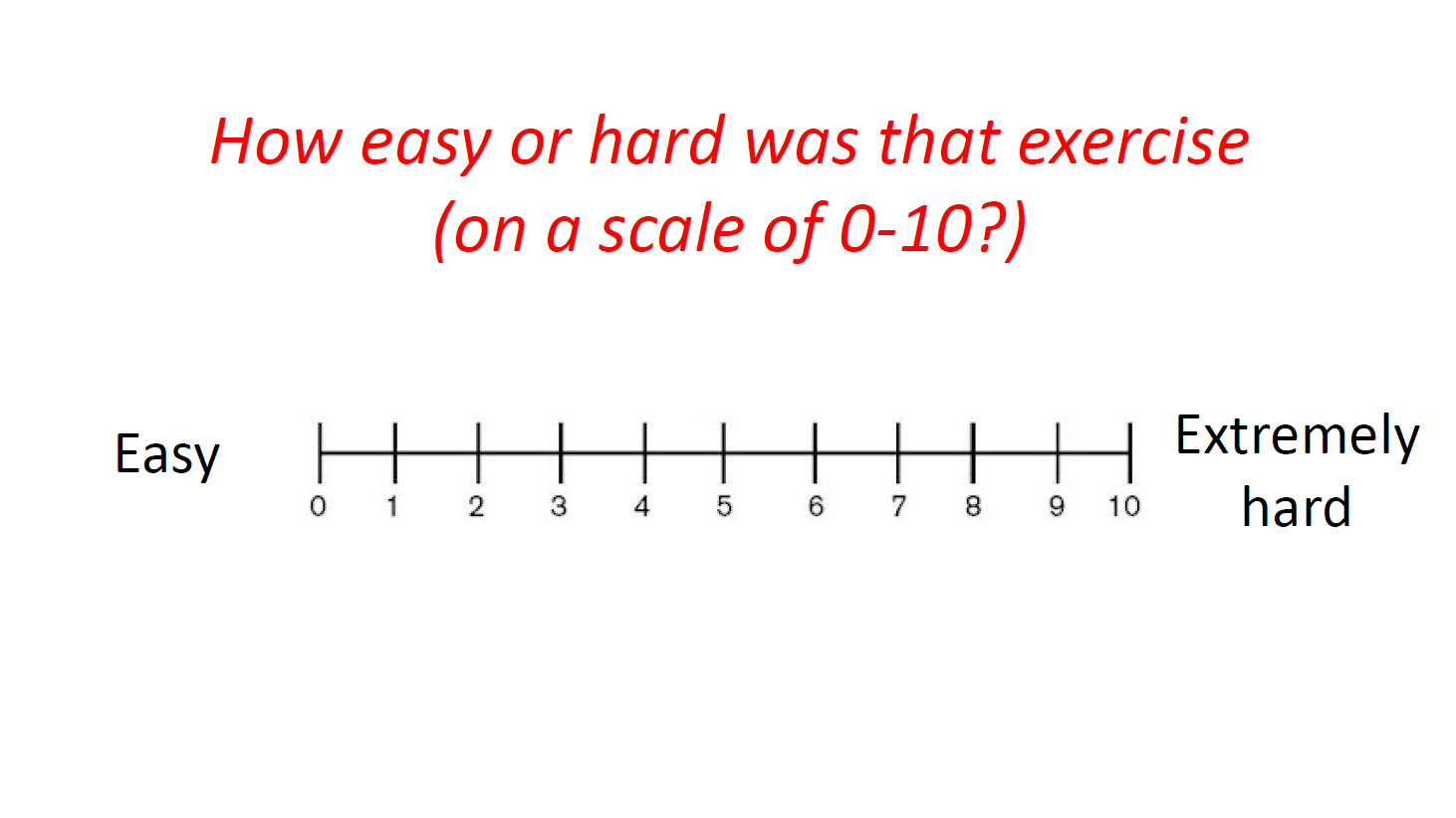


Supplementary figure 3: ‘easy, medium, hard’ picture word board.


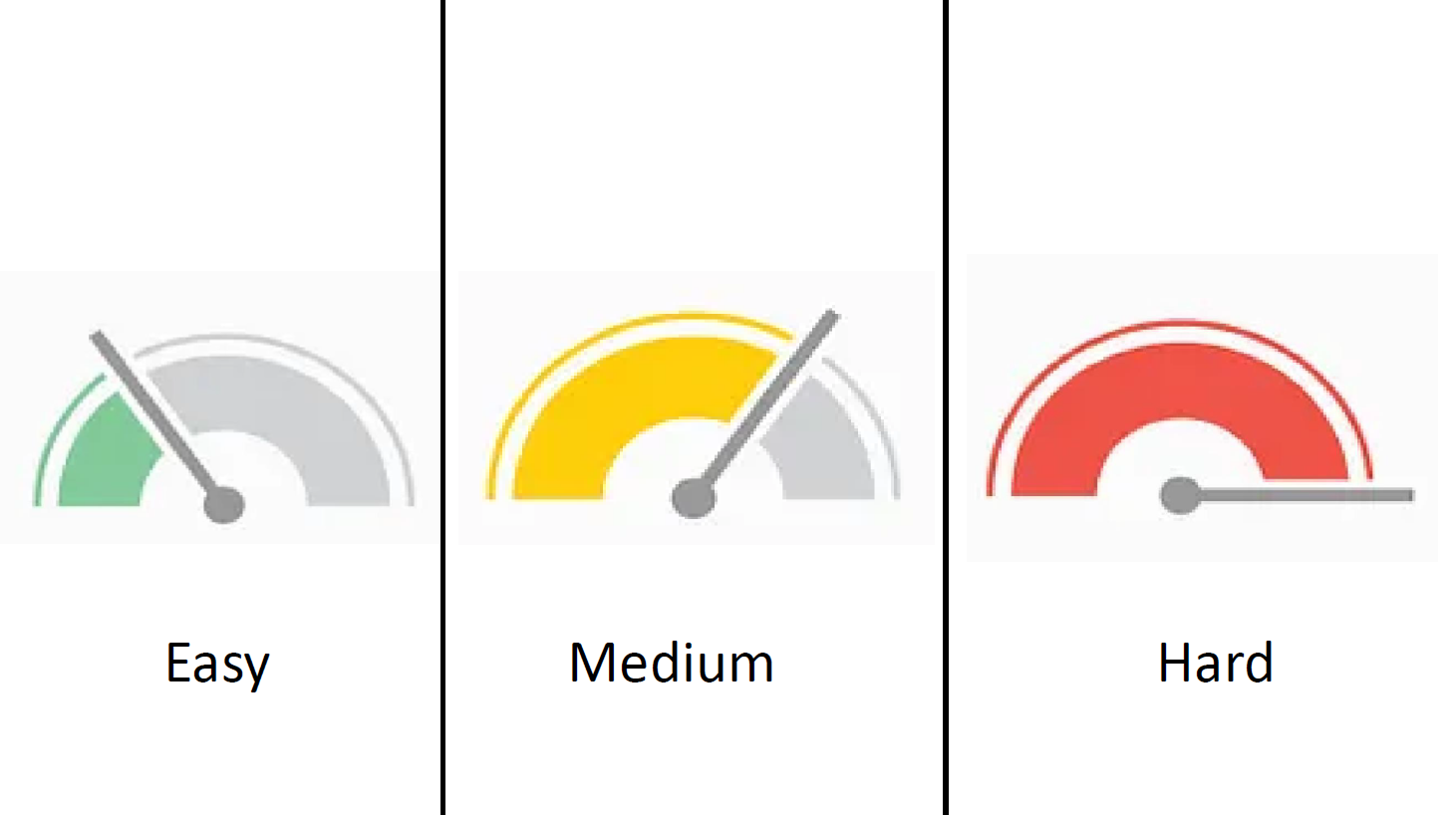


Supplementary figure 4: ‘pleasantness’ picture word board.


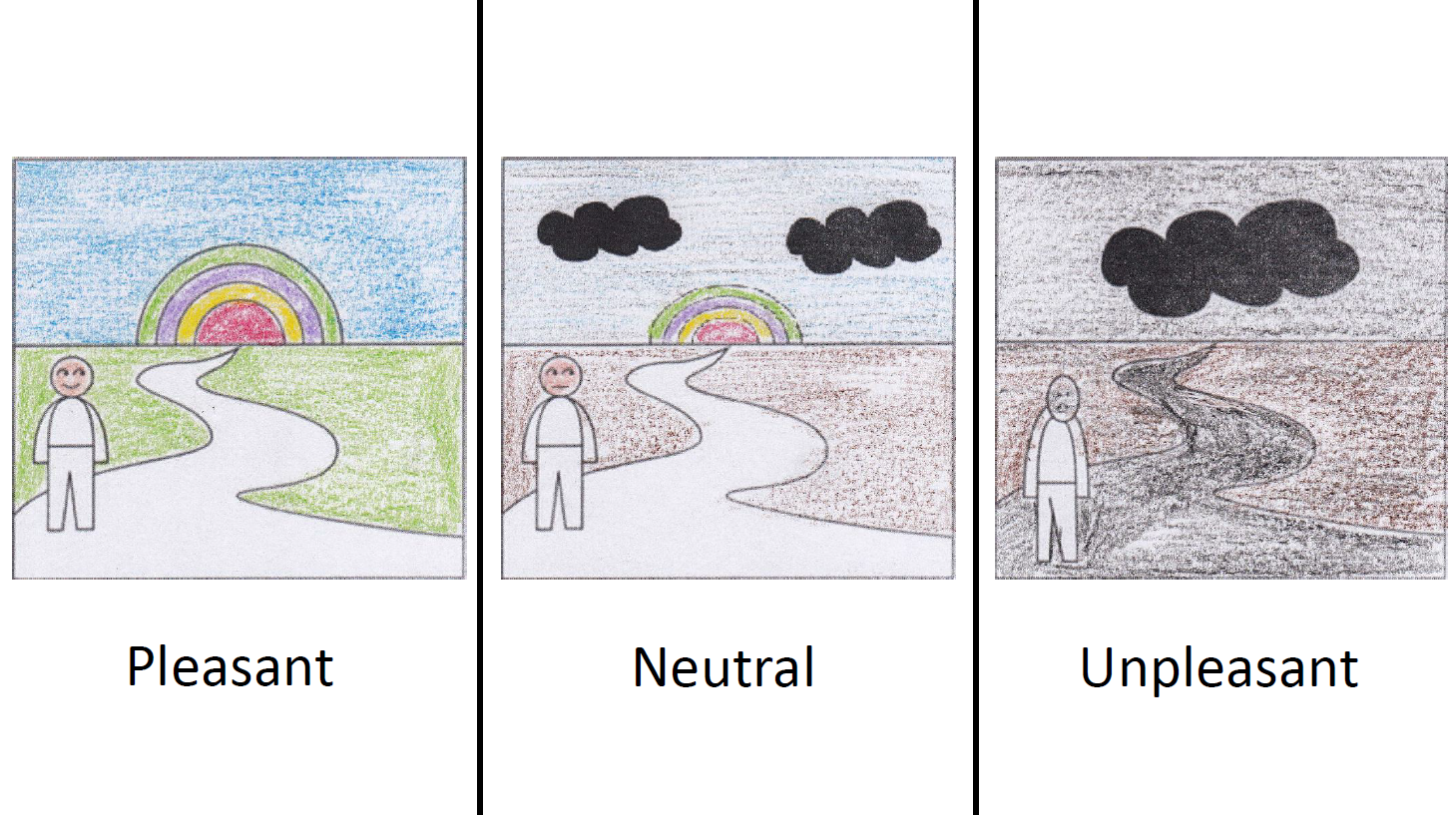


Supplementary figure 5: ‘feeling’ picture word board.


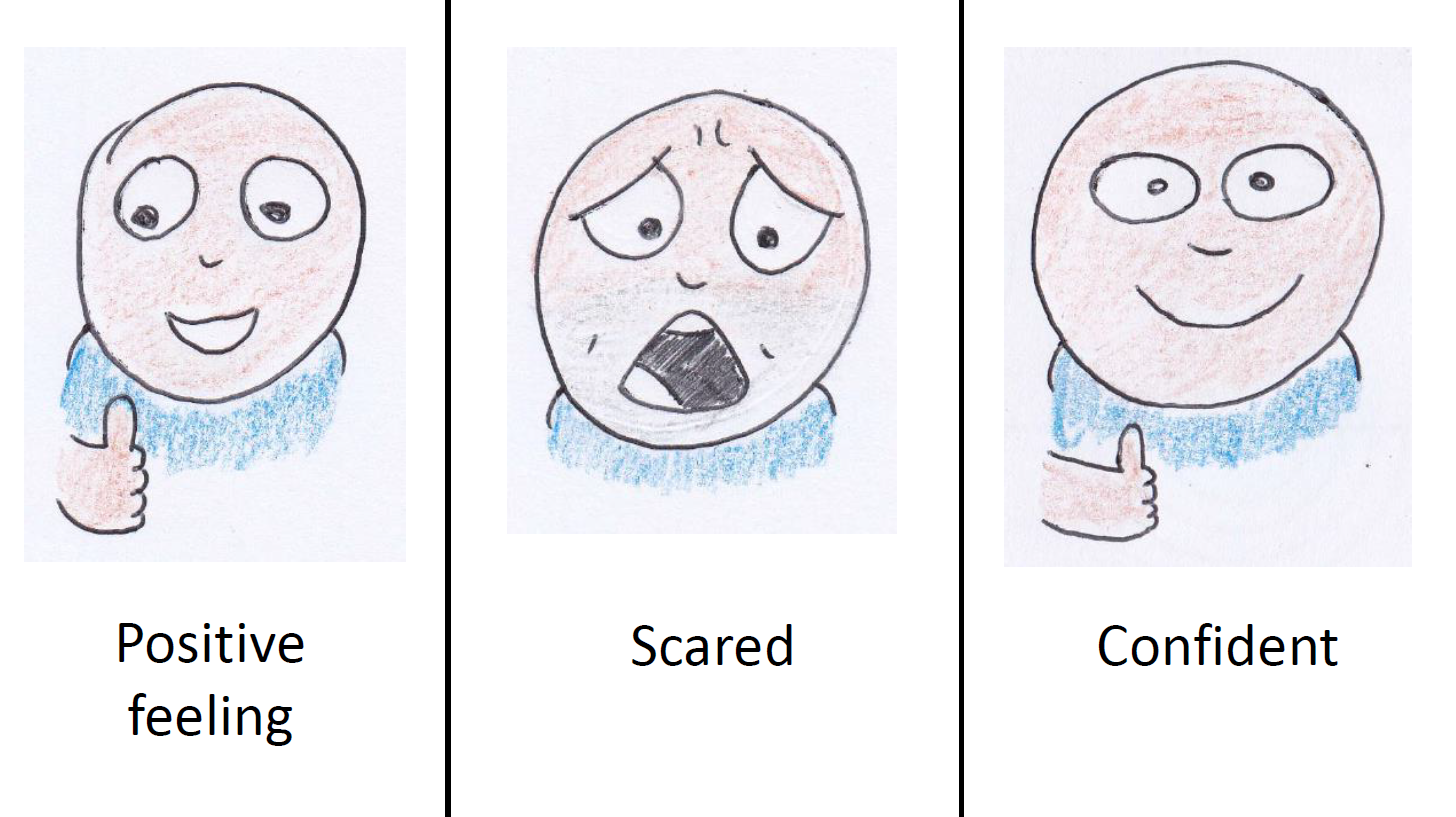


Supplementary figure 6: ‘experience’ picture word board.


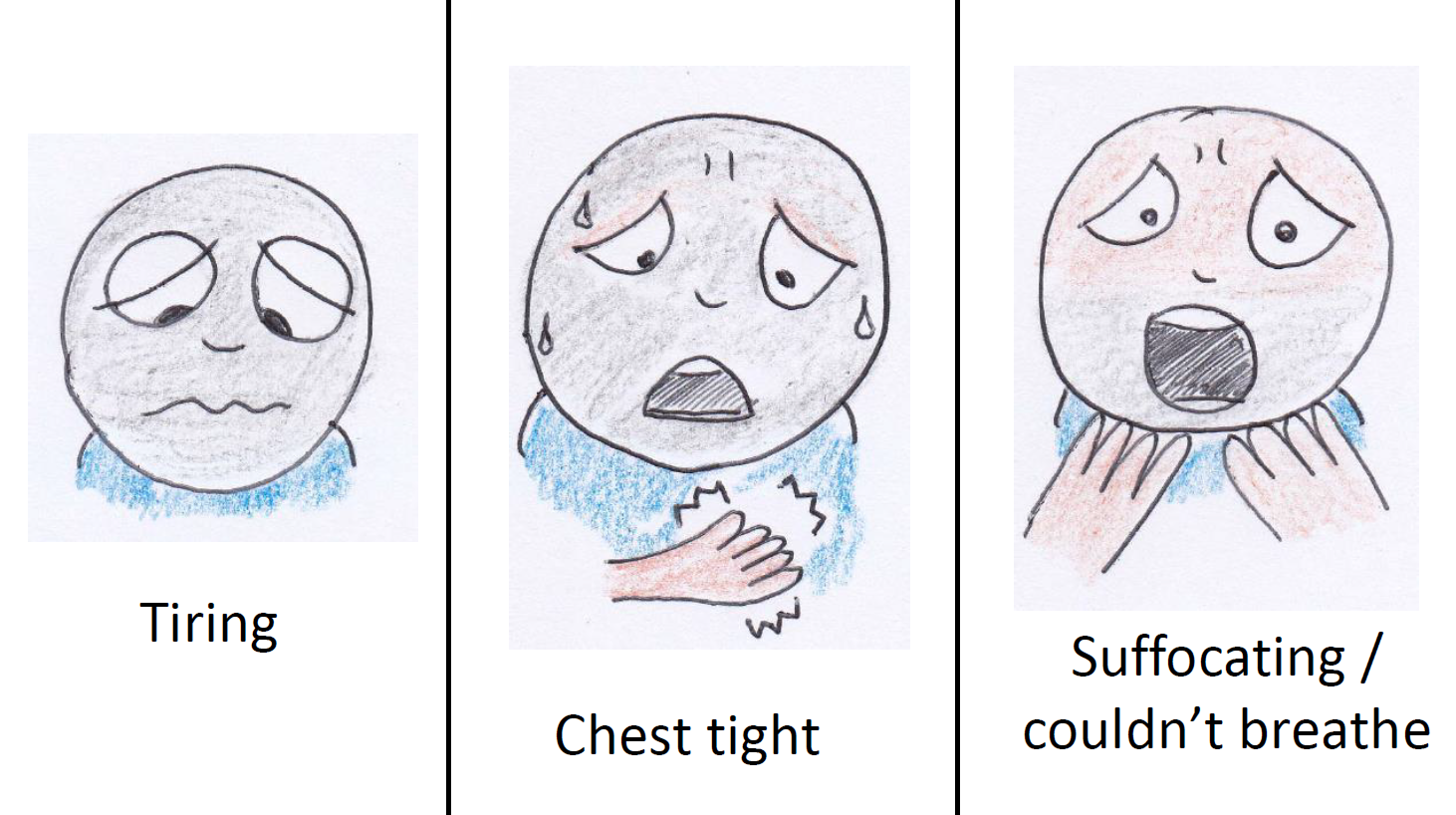

Supplement: online supplemental file 1 [file bmjresp-13-1-s001.docx]
